# Toward the Emergence of Intelligent Control: Episodic Generalization and Optimization

**DOI:** 10.1162/opmi_a_00143

**Published:** 2024-05-10

**Authors:** Tyler Giallanza, Declan Campbell, Jonathan D. Cohen

**Affiliations:** Department of Psychology, Princeton University, Princeton, NJ, USA; Princeton Neuroscience Institute, Princeton University, Princeton, NJ, USA

**Keywords:** cognitive control, context processing, concept formation, categorization, state space abstraction

## Abstract

Human cognition is unique in its ability to perform a wide range of tasks and to learn new tasks quickly. Both abilities have long been associated with the acquisition of knowledge that can generalize across tasks and the flexible use of that knowledge to execute goal-directed behavior. We investigate how this emerges in a neural network by describing and testing the Episodic Generalization and Optimization (EGO) framework. The framework consists of an episodic memory module, which rapidly learns relationships between stimuli; a semantic pathway, which more slowly learns how stimuli map to responses; and a recurrent context module, which maintains a representation of task-relevant context information, integrates this over time, and uses it both to recall context-relevant memories (in episodic memory) and to bias processing in favor of context-relevant features and responses (in the semantic pathway). We use the framework to address empirical phenomena across reinforcement learning, event segmentation, and category learning, showing in simulations that the same set of underlying mechanisms accounts for human performance in all three domains. The results demonstrate how the components of the EGO framework can efficiently learn knowledge that can be flexibly generalized across tasks, furthering our understanding of how humans can quickly learn how to perform a wide range of tasks—a capability that is fundamental to human intelligence.

## INTRODUCTION

Among the most striking features of human intelligence are the breadth of tasks we can learn to perform, the efficiency with which we can do so, and the flexibility with which we can generalize this learning to new tasks and switch among them. Although non-human animals and machines can outperform humans in many *particular* tasks (such as building webs or dams, doing arithmetic, or playing a variety of games from Jeopardy to Go), humans can achieve a respectable level of performance over a vastly broader range of tasks than any other known agent, natural or artificial. Furthermore, although machines are now superior on many tasks traditionally used to index intelligence (such as playing chess) and are approaching human competence on others (such as language generation), they generally require massive amounts of training experience to do so—far in excess of that required by humans—while exhibiting considerably more restricted ability to generalize that experience to other similar tasks (Lake et al., 2017).

It is generally agreed that the flexibility of human cognition reflects a core competency: the ability to efficiently acquire and represent knowledge in such a form that it generalizes effectively to tasks or situations that share similar structure. This idea unites cognitive psychology, cognitive neuroscience, and machine learning: work in cognitive psychology highlights the importance of abstraction and analogical reasoning in general intelligence (Holyoak, 2012; Newell & Simon, 1972); work in cognitive neuroscience suggests that transmodal and cross-task representations are critical for semantic decision making (Patterson et al., 2007) and cognitive control (Musslick et al., 2023); and work in machine learning demonstrates that multi-task learning and meta-learning greatly increase data efficiency in artificial neural networks (Caruana, 1997; Thrun & Pratt, 1998). However, it remains a mystery how people are able to acquire such abstract knowledge so efficiently from their experience and a challenge to design artificial systems that can emulate this ability.

In this article, we suggest an approach to addressing this mystery that builds on two lines of research in cognitive science often treated as distinct—the acquisition of knowledge and the execution of goal-directed behavior—integrating these in a way that leads to a reframing of core constructs in each.

### Long-Term Memory and Generalization

Work on the acquisition of knowledge has traditionally focused on mechanisms of learning and the organization of long-term memory (McClelland et al., 1995; Squire, 1987). This work has been shaped by the longstanding formulation of long-term memory as comprised of two components (Ebbinghaus, 1885; Tolman, 1948; Tulving, 1972): *episodic* memory, which refers to memory for individual events or experiences (such as what you ate for breakfast yesterday); and *semantic* memory, which refers to knowledge about the structure of the world, often framed in statistical terms (such as what *kinds* of items people *typically* eat for breakfast). The distinction between episodic and semantic memory has been identified as an important computational principle, that can help explain brain organization, and has driven tremendous progress in our understanding of human cognitive function and the neural mechanisms from which it arises (Kumaran et al., 2016; O’Reilly et al., 2014). Complementary Learning Systems (CLS) theory formalizes this distinction in terms of a fundamental tradeoff in neural network architectures between the ability to rapidly acquire new knowledge, often requiring the formation of novel associations among (seemingly) arbitrary pieces of information in episodic memory, while preserving (i.e., not overwriting) existing knowledge about structure in the world acquired through the integration of information in semantic memory over extended experience (McClelland et al., 1995).

One general implication of the CLS formulation has been that the episodic memory system is not directly involved in generalization, as it is responsible for encoding specific individual experiences, rather than general patterns that hold across many experiences which are assumed to be learned by slower, statistical learning mechanisms in semantic memory.^1^ However, the ability to rapidly encode episodic memories in a novel circumstance and recall these memories later to guide inference or action in new but similar situations may also provide a means for generalizing across experiences. The instance-based nature of the memories means this process can be extremely data-efficient: making an inference requires, in the limit, recalling just a single memory of a related prior experience (e.g., inferring that a person you recently met will eat oatmeal for breakfast today because they ate oatmeal for breakfast yesterday).

### Cognitive Control and Working Memory

While the considerations above suggest that episodic memory plays an important role in the rapid adaptation of behavior, such work has focused primarily on memory rather than action; most research addressing the flexibility of task performance has focused on mechanisms underlying cognitive control and its use of working memory. Cognitive control refers to the updating and maintenance in working memory of representations of task demands, goals, or contextual factors that are used to guide processing to align with the current situation (Cohen, 2017; Salamé & Baddeley, 1982). Control is important in quickly changing environments, and in particular when a stimulus demands different, and especially less habitual responses that are specific to the situation. Under these conditions, it has been proposed that the role of control is to limit interference among conflicting signals, allowing a single system to operate in a wide range of situations without such interference (Botvinick et al., 2001). For example, in the Wisconsin Card Sort Task participants must sort cards according to the shape, color, or number of symbols appearing on the card, with the sorting rule changing periodically (Berg, 1948). Maintaining a representation of the current rule (e.g., sort by color) and using it to influence processing (e.g., by attending to color) ensures that only context appropriate responses are made (e.g., it prevents accidentally sorting by number, even if that rule was recently active; Miller & Cohen, 2001).

Despite the centrality of control representations to theories of control, little work has focused on the structure of these representations or how they are learned. Recent work has begun to address this question, suggesting that the statistical learning mechanisms responsible for the formation of representations in semantic memory may be critical not only for learning about the structure of the world, but also how to behave within it, shaping the representations used for the control of behavior much as it shapes the representations used to understand the world itself (Giallanza et al., 2023). To date, however, little work has considered whether and how the capabilities afforded by episodic memory may contribute to the formation and use of such representations; that is, how episodic memory may interact with cognitive control.

### Episodic Memory and Control

**Figure 1.**
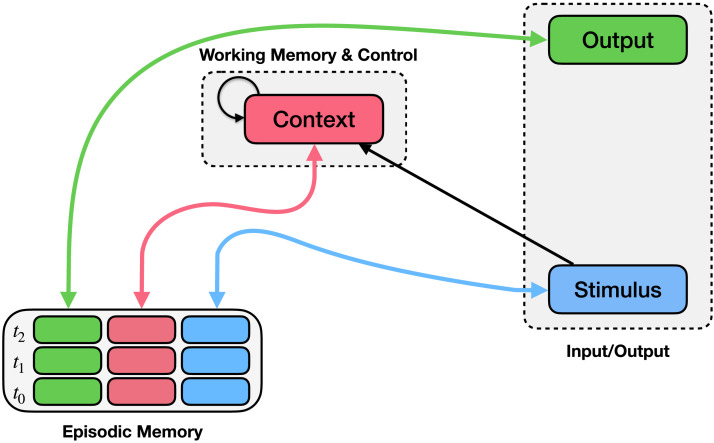
**The Episodic Generalization and Optimization (EGO) Framework for Studies 1 & 2.** The EGO framework consists of a control mechanism (context module; upper middle) and an episodic memory mechanism (bottom left). Episodic memory records conjunctions of stimuli (blue boxes), contexts (pink boxes), and observed responses (green boxes) at each time point (rows). Bidirectional arrows connect episodic memory to the stimulus, context, and output, indicating that these values can be stored in or used to query episodic memory, or retrieved from it when another field is queried. The context module integrates previous context (recurrent connection) along with information about the stimulus and the context retrieved from memory.

In this article we consider these interactions in detail, by examining how learning and episodic memory contribute to the flexibility of control, enabling rapid generalization and the optimization of behavior for a task. Specifically, we show that storing information in episodic memory and retrieving that information in the future enables the rapid and efficient transfer of knowledge useful for control from previously experienced situations to new ones. Conversely, control can help avert a potential cost in the use of episodic memory: retaining old memories runs the risk of creating confusion when old knowledge conflicts with new information. In these cases, control can be used to intervene by guiding the memory retrieval process, providing context that disambiguates otherwise conflicting memories and aids recall of task-appropriate ones. In combination, episodic memory and control work to store memories of past experiences and the context in which those experiences occurred, accessing memories from similar situations in the past that may be useful in the current situation. This idea builds on previous suggestions that the *binding* function of episodic memory (McClelland et al., 1995) and the *biasing* effect of context representations on processing (Cohen et al., 1990) can be viewed as two complementary forms of control (Cohen & O’Reilly, 1996). Here we suggest that, working together (as shown in Figure 1), these may help explain both the rapid learning of new tasks and the breadth of tasks that can be performed.

We formalize these ideas and explore them using computationally explicit mechanisms in what we refer to as the Episodic Generalization and Optimization (EGO) framework, which implements episodic memory and context-dependent control of processing within a standard neural network architecture. This is inspired by previous work exploring the relationship between episodic memory (as a form external memory) and recurrent neural networks used for control (e.g., Fortunato et al., 2019; Graves et al., 2014; Ritter et al., 2018; Webb, Frankland, et al., 2023; Webb et al., 2020). Here, we use this framework to address empirical phenomena across three domains of function that have largely been treated separately of one another (reinforcement learning, event segmentation, and category formation), showing in simulations that the same underlying mechanisms can account for characteristically human patterns of behavior in these different domains. Specifically, the simulations describe how episodic memory and context-dependent control can enable human-like inference in sequential prediction tasks, how the control system learns to segment continuous experience to disambiguate conflicting memories, and how episodic memory bootstraps the learning of abstract context representations used to control inference and behavior in category learning. We use the simulations to illustrate the factors of the environment and the system that contribute to generalization, such as the temporal structure of experience and the specificity versus generality of memory retrieval, providing an integrated account of findings previously addressed separately by models of reinforcement learning, episodic memory, and cognitive control.

## THE EGO FRAMEWORK

We begin by providing an overview of the modeling framework. The EGO framework consists of two key components that interact to produce context-appropriate responses to stimuli given a small amount of training data: an episodic memory mechanism (lower left component of Figure 1) and a control mechanism (upper middle component of Figure 1), each of which is implemented using standard components of neural network architectures from prior work. We begin by presenting a simplified version of the framework used to construct the model of Study 1. In Studies 2 and 3, we build upon this foundation, expanding the framework slightly to accommodate more complicated tasks.

### Episodic Memory Mechanism

The episodic memory mechanism records a history of encountered data and is implemented as a differentiable external memory (Fortunato et al., 2019; Graves et al., 2014; Ritter et al., 2018; Webb et al., 2020). It represents memories using three matrices: one for stimulus information, one for context information, and one for output information (e.g., the reward in reinforcement learning tasks or the response in stimulus-response association tasks). Each time-point in the simulation is associated with a row in these three matrices, and writing to episodic memory occurs after each stimulus presentation: a new row is appended to memory that contains the new stimulus, the new context, and the outcome associated with the new stimulus (in other words, new memories are always written to new rows, as in Fortunato et al., 2019 and Webb et al., 2020, but contra Graves et al., 2014).

Memory is queried every time a new stimulus is presented. When a new stimulus is presented, that stimulus and the current context are compared to stimulus/context pairs in memory based on a similarity function (i.e., cosine similarity between the vector representations), providing a score indicating how well each memory matches the current situation. Taking a weighted average of past memories (weighted by the match score) results in a retrieved memory, containing a retrieved stimulus, retrieved context, and retrieved outcome; this information guides behavior by providing examples of outcomes that occurred in similar situations in the past (similar to exemplar-based models of categorization; Nosofsky, 1986; and kernel-based methods in machine learning; Boser et al., 1992; Vapnik & Lerner, 1963).

For example, consider using the episodic memory mechanism to help infer how much you will enjoy eating a new dish at a restaurant. First, the context (the restaurant) and the stimulus (the new dish) are compared to previous experiences in memory. This results in a score indicating how similar each memory is to the current situation. These scores are then used to weight past experiences, favoring those that involve similar dishes and/or similar restaurants to the current situation; by averaging over how much you enjoyed each previous situation, the system makes an inference about how much you will enjoy the current one.

### Control Mechanism

As mentioned in the previous section, memory encoding and retrieval in the EGO framework is coordinated by a representation of the current context. For the first study, we implemented context as a linear integrator over the stimuli. On every timestep, the controller integrates the context from the previous timestep with both the context retrieved from episodic memory and the current stimulus to form the new context. In so doing, the context represents the sequence of events leading up to, and the stimuli present in the environment during, performance of a particular task.

This use of linear integration as a form of context follows the traditional view of context in models of episodic encoding and recall (e.g., Tulving, 1972) and, in particular, is closely related to the TCM model (Howard & Kahana, 2002; Polyn et al., 2009). Recent work has suggested that this form of context can not only account for patterns of human behavior in free recall tasks, but also provide a useful form of context for sequential learning problems by providing a representation of the transitions between states in the environment (Zhou et al., 2023). In Studies 2 and 3, we modify the control mechanism to handle more complicated forms of context that require non-linear integration.

### Summary

In summary, the EGO framework describes how episodic memory and cognitive control can work together to enable flexible processing and inference in novel environments. The key component is the coupling of standard mechanisms of learning with context-dependent re-use of previous experiences through memory recall. This provides a data-efficient form of learning, explaining humans’ remarkable ability to use previous experience to make new inferences and to learn new behaviors with far less data than most existing neural network architectures.

In the remainder of this article we apply the framework to simulate key phenomena observed in three different lines of empirical inquiry that have previously been explained using different formalisms: 1) the effects of reward versus transition revaluation in Momennejad et al.’s (2017) multi-step learning task, explained in terms of combination of model-based decision making (Daw et al., 2011) and the learning of successor representations (Dayan, 1993; Gershman et al., 2012) in prediction and decision-making; 2) the effects of blocked versus interleaved training in Beukers, Collin, et al.’s (2024) state prediction task, explained in terms of latent cause inference (Courville et al., 2006) for event segmentation in the context of continual learning; and 3) the effects of blocked versus interleaved training on the structure of and selection among semantic representations formed in Flesch et al.’s (2018) categorization task, explained in terms of a variant of Hebbian learning. We show that interactions between context processing and episodic memory are sufficient to reproduce the phenomena in each of these domains, providing a unifying account in terms of a single, integrated set of underlying learning and processing mechanisms.

## STUDY 1: REINFORCEMENT LEARNING AND DECISION MAKING

### Rationale

**Figure 2.**
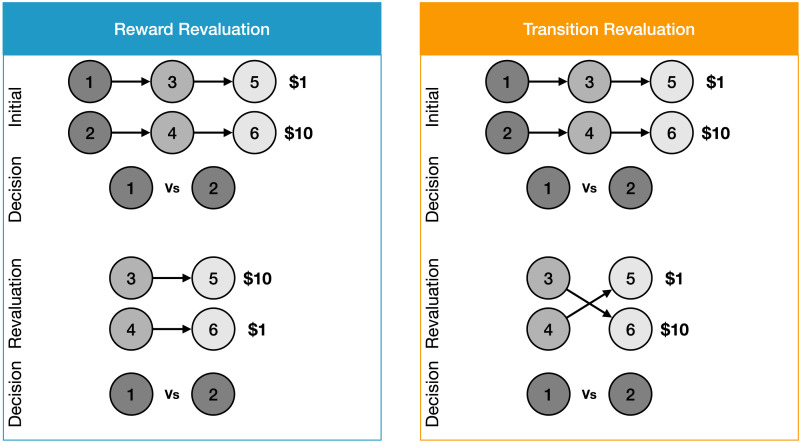
**Sequential learning task (Momennejad et al., 2017) used for Study 1.** Flow of the reward and transition revaluation conditions. The graphs show the trajectories in the two conditions. Both conditions start with the same transition structure in the learning phase. In the revaluation phase, either the rewards (left) or transitions (right) in each trajectory change. At the end of the learning and revaluation phases, participants and the model decide which starting state they prefer (state 1 or state 2) based on their predictions regarding the trajectories and rewards associated with the final states.

We first demonstrate the framework’s ability to explain the flexibility of human-like inference in a changing environment by simulating the multi-step learning task introduced by Momennejad et al. (2017). This task is based on the two-step task in Daw et al. (2011) that has been used extensively to study the relative contributions of model-based versus model-free reinforcement learning to prediction and decision making (Daw et al., 2005). The task tests the extent to which an agent can flexibly adapt to two kinds of changes in the environment: reward revaluation and transition revaluation (Figure 2).

### Behavioral Task

In an initial learning phase, participants were shown two different sequences of three stimuli, each followed by a different reward value. In a second, revaluation learning phase, participants were shown sequences of two stimuli, each beginning with the second stimulus in one of the sequences seen in the initial learning phase, followed by one of two changes. In the *reward revaluation* condition, the sequence continued as in the learning phase, but the reward associated with the two trajectories was swapped. In the *transition revaluation* condition, the third stimulus was swapped between the two sequences, which was then followed by the same reward that originally followed each of the third stimuli in the learning phase. Finally, in a decision phase that followed each revaluation learning phase, participants indicated their preference between the starting states of the two trajectories (i.e., the first stimulus seen in the initial learning phase), providing a measure of how effectively participants were able to adapt their preference following the revaluation.

Both revaluation conditions have traditionally been assumed to index the contribution of model-based (MB) learning (Daw & Dayan, 2014; Daw et al., 2005; Sutton & Barto, 2018), as they involve a change in *distal* reward: in the final decision phase, participants choose between the starting states of the trajectories, which were not re-experienced in the revaluation phase, so the task cannot be learned using standard model-free (MF) reinforcement learning. However, MB learning can be used to perform the task by representing the transition from each stimulus to the next as well as the reward associated with each stimulus, updating these when it encounters new information. This suggests that once a fully MB agent has experienced all possible states, transitions, and rewards, it should perform perfectly following revaluation.

Momennejad et al. (2017) found that participants performed well above chance in both revaluation conditions, indicating that they were not relying exclusively on MF processing. However, their performance fell substantially short of perfect, suggesting they also did not rely entirely on MB processing. One possibility is that performance reflected a mixture of MB and MF processing. However, another subtle but significant finding was that performance was slightly better for reward revaluation than for transition revaluation. This is not easily explained by a simple mixture of MB and MF processing. Instead, the authors proposed that participants used a mix of MB processing and successor representation (SR) learning (Dayan, 1993; Gershman et al., 2012). SR involves the use of MF learning to build an internal representation of the transitions among stimuli and a separate representation of the reward associated with each stimulus. An SR agent performs well on reward revaluation because it can easily update its reward-to-stimulus mapping; but it fails entirely on transition revaluation because updating its transition representation requires directly experiencing every full sequence of transitions (e.g., the trajectory 1 -> 3 -> 6 to learn the association between states 1 and 6).

Momennejad et al. (2017) showed that a mixture of MB processing and SR learning can reproduce the pattern of behavioral effects they observed. Here, we show that a model based on the EGO framework can also reproduce the empirically observed effects, which are explained in terms of a single set of interacting mechanisms: a continuously integrated context representation and the use of similarity-based retrieval of these context representations and previous experiences to make predictions from current stimuli. In the discussion, we consider the ways in which this account is both similar to but also differs from the SR/MB account.

### Model

We modeled this task using the EGO framework as described in Figure 1. We simulated the experiment by providing the model with the same data presented to the human participants in the Momennejad et al. (2017) study, representing each stimulus (e.g., each state in the sequence) with a one-hot encoding (see Supplementary Information for more details). The model was used to simulate human performance by executing in two modes: an observation mode, used to simulate the learning phases during which participants passively observed the state transitions; and a decision mode, used to simulate the decision phases during which participants estimated the value of the states.

**Figure 3.**
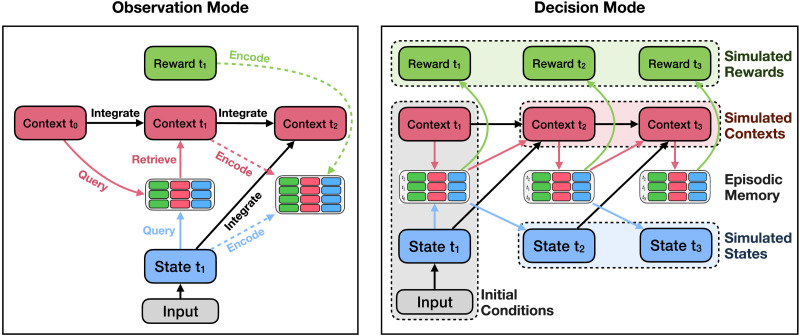
**Processing in observation and decision mode for Study 1.** In observation mode, the model first uses its context representation and the current stimulus to retrieve a memory. It uses the retrieved context to update the current context, then encodes the updated context representation, the current stimulus, and the observed reward provided by the environment into a new episodic memory. Finally, it integrates the current stimulus into its context representation. In decision mode, the model first uses one of the two starting states and its prior context to query episodic memory. It adds the retrieved reward to an estimate of the value of the initial state and uses the retrieved state and context to update its context representation. It then uses its new context representation to query memory again, adding the retrieved reward to its value estimate, and repeats this process until a termination condition is met.

**Figure 4.**
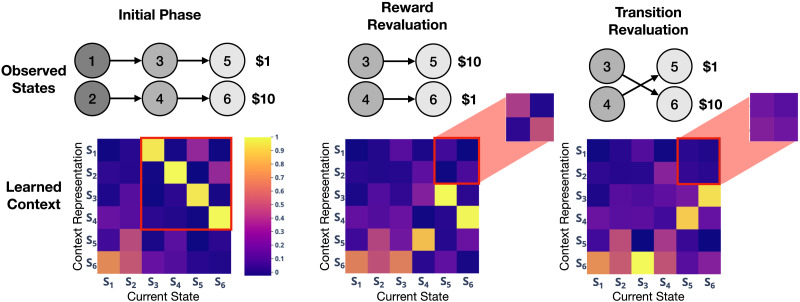
**Context representations for Study 1.** *Left*: Context representations learned during the initial phase, averaged over memories for each state. Columns show the context representation for each state—for example, the context representations for state 5 reflect that state 3 is the one-step predecessor of state 5 (yellow square) and state 1 is the two-step predecessor (pink square). *Middle*: Context representations during the reward revaluation phase. The context representations for states 5 and 6 still reflect the two-step dependencies (inset). *Right*: Context representations during the transition revaluation phase. The one-step transitions for states 5 and 6 update following transition (yellow squares in the center right of the heatmap), but the two-step transitions are no longer represented (inset).

In observation mode (Figure 3, left side), the model first observes the current stimulus. It then uses the state and the current context to query episodic memory, retrieving a memory of a prior context associated with the state and integrating this context retrieved from episodic memory into the current context. Next, the model observes the reward associated with the state, storing the state, the updated context, and the reward as a new row in episodic memory. Finally, it updates the context once again by integrating the representation of the current state. It then repeats this process with the next state/reward pair. This results in the context representations encoding the long-run discounted transitions between the states, as the context at time *t* encodes a recency-weighted representation of previously visited states (Figure 4; see also Gershman et al., 2012).

In decision mode (Figure 3, right side), the model simulates a future trajectory to estimate the long-term reward for a given state by iteratively updating its context representation and querying episodic memory. Given a starting state, the model first queries episodic memory using the state and the current context, resulting in a retrieved state, retrieved context, and retrieved reward. It then updates its context using the retrieved state and context, using the updated context to once again retrieve a memory. The model then repeats this process until a termination condition is met (e.g., it makes a fixed number of queries), resulting in a sequence of recalled memories that, since the context representation integrates information about prior states, respect the previously observed transitions between states (see also Shohamy & Daw, 2015). The model can then estimate the long-run value of the starting state by summing over the simulated rewards. As in the behavioral experiment, the model can run the value estimation process for two different states (i.e., State 1 and State 2, the starting states) and choose which state it prefers by comparing the values.

### Results

**Figure 5.**
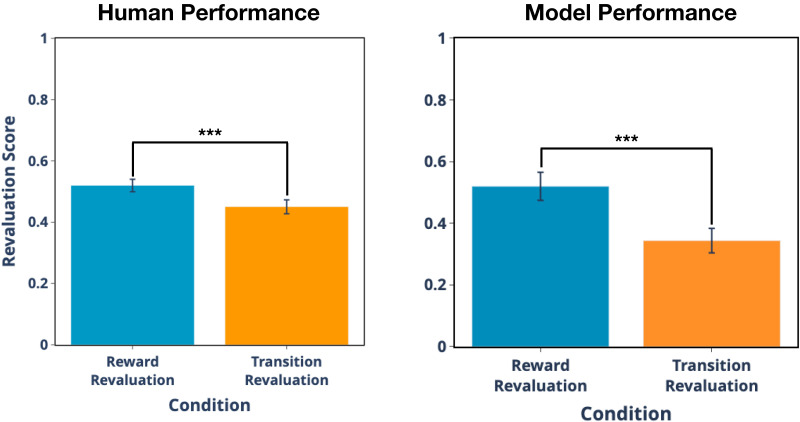
**Results for Study 1.** Revaluation scores measure the change in preference ratings for the starting states before versus after revaluation. For both the model and humans, revaluation scores are greater for reward revaluation than for transition revaluation.

We simulated performance of the task and compared the model’s behavior to the human behavior reported in Momennejad et al. (2017). We exposed the model to the same number of trajectories as participants, running the model 58 times with random initial conditions to simulate performance of the 58 human participants in the empirical study. Following Momennejad et al. (2017), we quantified performance with a *revaluation score*, indicating the change in preference between the starting states before and after the revaluation phase (Figure 5). A value of 0 indicates no change in preferences while a value of 1 indicates a complete preference reversal.

The main result is that both human participants and the model update preferences following revaluation, with revaluation scores significantly greater than 0 (model: 0.52 ± 0.04 s.e.m. for reward revaluation and 0.34 ± 0.04 for transition revaluation; humans: 0.52 ± 0.02 for reward revaluation and 0.45 ± 0.02 for transition revaluation; Figure 5). Additionally, the mean revaluation score was modestly but significantly larger in the reward revaluation condition than in the transition revaluation condition (by unpaired *t*-test for the model: *t*_114_ = 4.050, *p* < .0001; and by paired *t*-test for humans: *t*_57_ = 10.148, *p* < .0001).

### Discussion

The pattern of the model’s performance arises from the interaction between the control and episodic memory mechanisms, which in combination implement key features of both MB and SR learning algorithms. Like MB, the model estimates the value of a state by simulating step-by-step “rollouts” of future trajectories from that state. However, it does so differently than traditional model-based algorithms. While MB explicitly represents a stimulus-to-stimulus transition matrix and a stimulus-to-reward matrix, updating values in these matrices as they change, our model *implicitly* represents these by maintaining unchanging copies of all previously experienced stimuli, contexts, and rewards in memory. It then accesses these memories using a representation of the current context: by encoding the context in which a state occurred (e.g., that state 3 came after state 1), and by querying with the relevant context when running a simulation (e.g., querying memory with a context encoding state 1, and retrieving a memory of state 3), the model accesses memories in an order that respects the observed transitions in the environment.

In contrast to the MB approach of using a veridical lookup from a transition matrix to determine the next simulated state, our memory-based approach uses a stochastic match between the current context and remembered contexts, and this stochasticity occasionally results in retrieving outdated memories (i.e., memories from the initial phase, such as state 5 yielding $1, that contradict with updated memories in the revaluation phase, such as state 5 yielding $10). This explains why our model, like humans, does not fully update its preferences after the revaluation phase (i.e., it has a revaluation score of less than 1; see Figure 5) and suggests that the sub-optimality of MB processing observed in humans could be due to errors in *retrieval* rather than, or in addition to, failures of learning and/or encoding of associations.

A further distinction from MB is that the context representations the model uses to retrieve memories encode *long-term* transitions between states. This biases the simulation mechanism to favor retrieval of memories consistent with not only the one-step transitions but also the multi-step transitions between the starting state and subsequent ones. For example, if the model has simulated the trajectory 1 -> 3 following transition revaluation and needs to predict the next state, the two-step transitions will bias the model towards retrieving the outdated memory that the next state is state 5 rather than the updated memory that the next state is state 6. This is because the context associated with state 5 in the initial phase encodes a two-step association with state 1 (Figure 4, left panel), while the context associated with state 6 in the revaluation phase only encodes the one-step association to state 3 (Figure 4, right panel). While the influence of the two-step transitions is relatively weak compared to the associations encoded between one-step transitions, it is sufficient to provide a small but significant amount of interference at retrieval. In contrast, in reward revaluation the context representations continue to encode the (unchanged) two-step association between states 1 and 5 throughout the revaluation phase (Figure 4, middle panel), resulting in better performance compared to transition revaluation.

The two key distinctions between our model and conventional MB algorithms mentioned above—that our model retains old memories instead of updating them, leading to occasional retrieval errors, and that our model retrieves memories based on multi-step transitions, harming its performance following transition revaluation—both reduce the performance of the model on this task. In different environments, however, these features can be advantageous, providing flexibility not afforded by conventional MB processing in two closely related ways. First, the sensitivity of the model to multi-step dependencies can help performance on tasks that require maintaining information over an extended delay. For example, the AX-CPT task from the cognitive control literature requires responding to the letter “X” in one way *unless* it follows the letter “A”, in which case the response is different (i.e., it requires treating the sequence ? -> X, where “?” represents any letter other than “A”, as different from the sequence A -> X; Cohen & Servan-Schreiber, 1992; Todd et al., 2008). This distinction corresponds to the different trajectories in the learning and revaluation phases of the Momennejad et al. (2017) experiment (e.g., the sequence 1 -> 3 occurs in the initial phase, while the sequence ? -> 3 occurs in the transition revaluation phase); however, in the case of the AX-CPT task, sensitivity to the multi-step transitions is advantageous rather than a hinderance as it allows the agent to learn distinct, context-sensitivity responses to the same stimulus.

Second, retaining memories in episodic memory supports the recovery of previously experienced state dynamics. This corresponds to reinstatement (or “renewal”) effects in extinction learning paradigms: when humans or non-human animals are trained on an association, followed by a period in which the association is absent or violated, they quickly adapt to a reinstatement of the initial association (Bouton & Bolles, 1979; Pavlov, 1927; Phelps et al., 2004). More generally, humans appear to naturally segment continuous experience into chunks of recurring sequences of states and/or actions, often referred to as “events” or “schemas” in cognitive psychology (Newtson & Engquist, 1976; Rumelhart, 1975; Schank & Abelson, 1977), or “options” when this involves useful sequences of actions in the context of hierarchical reinforcement learning (Sutton et al., 1999). A number of studies of such “event segmentation” have revealed characteristic patterns of performance that are distinct from those observed for standard neural network learning but that are well characterized by probabilistic models of inference. In the section that follows, we consider such paradigms, showing again that a model cast within the EGO framework can reproduce these patterns using a psychologically and neurally plausible set of processing mechanisms that exhibit many of the same features as existing, more abstract models.

## STUDY 2: SEQUENCE LEARNING AND STATE SPACE ABSTRACTION

The previous study brings into focus both advantages and disadvantages of the storage and context-guided retrieval of memories in episodic memory: On the one hand, this allows for generalization between related memories, such as learning multi-step (non-Markovian) dependencies between stimuli and retrieving old memories when they become relevant. On the other hand, this creates the potential for interference between conflicting memories, risking retrieval of irrelevant memories that interfere with task performance. This tradeoff presents an optimization problem, balancing the benefits of retrieving relevant memories in the service of cross-memory generalization against the risk of cross-memory interference posed by the retrieval of irrelevant memories.

Context is critical in mediating this trade-off by favoring the retrieval of memories that have context representations similar to the current one. This suggests that if the system represents context similarly for related memories but differently for unrelated memories, it can guide retrieval to more strongly favor those that are relevant in the current situation. Here, we investigate two factors influencing the development of such context representations: the order in which stimuli are experienced in the environment, and its interaction with the use of learning to extract useful context information from the environment.

### Rationale

As demonstrated in Study 1, and consistent with TCM, recurrently integrating the previous context along with current stimulus information into the context representation influences the similarity between memories, such that memories occurring nearby in time have similar context representations. This implies that the order in which stimuli are experienced should affect the performance of the model: The model should be better able to retrieve relevant memories and should thus perform better overall when related stimuli appear clustered together in time (i.e., in a blocked design) than when related stimuli are more separated in time and/or unrelated stimuli are closer together in time (i.e., in an interleaved design).

Although the integration of context representations may be useful in general, given that information in the environment is often temporally autocorrelated (Zacks & Tversky, 2001), the simple form of linear integration used in Study 1 may not always be the most efficient or useful way to impose structure on the context representations. Rather, in some circumstances it may be helpful to extract context-indicative information from the environment and use this to shape the internal representations of context through learning. For example, when watching a movie a change in the background can be a strong indicator of a change in the scene, and retrieving information from memory associated with the new background may help appropriate interpret and/or predict what happens next (Baldassano et al., 2017). Accordingly, learning how to use changes in context-indicative information to shape context representations may help the system better organize its memories to support future, task-appropriate behavior. Such shaping of context representations may be particularly useful when two situations strongly conflict with one another.

For example, many situations share similar stimuli but have different transition structures (e.g., a customer at a restaurant sits at a table and then orders food, whereas a customer at a food court orders food and then sits at a table). Simple linear integration will help the system learn the transition structure (as in Study 1), but it will only provide a weak cue about the difference between them due to the similarity between the situations. This can be overcome by further differentiating the context representations associated with each setting (e.g., learning distinct context representations for restaurants and food courts and using these contexts to guide retrieval of relevant memories). Recent empirical work suggests that people can learn how to do this very effectively, but that this depends on the temporal structure of the environment: people do better when trained in blocks of each situation than when trained interleaved (Beukers, Collin, et al., 2024).

One approach to explaining this uses Latent Cause Inference (LCI; Gershman et al., 2014) to assign context representations that help distinguish between the different transition structures (Beukers, Collin, et al., 2024). Here, we show how an extension of the model used in Study 1, in which the context layer is subject to learning, can provide similar functionality and, accordingly, a similarly accurate account of the findings. In the Discussion, we compare this model with LCI-based accounts and consider closely related issues such as the tension between pattern completion and pattern separation addressed by Complementary Learning System theory (McClelland et al., 1995).

### Behavioral Task

We simulated the continual learning experiment described in Beukers, Collin, et al. (2024). This experiment used a “next-state prediction task” to test participants’ ability to learn two sets of sequences involving an identical set of states presented in different orders. Each set of sequences was defined as a graph, with nodes corresponding to stimuli (states) and edges determining the transitions from one state to the next (see Figure 6). Participants were shown a series of states one at a time, determined by one of the graphs. Without being told about the sequential structure of each graph, they were periodically asked to predict the next state. As shown in Figure 6, each state (e.g., state 1) lead to one of two different successor states (e.g., state 3 or state 4), depending on which graph was currently in effect. Since state transitions depended on both the current state and the current graph, accurate performance required both inference of the graph structure and determination of which graph was currently in use.

**Figure 6.**
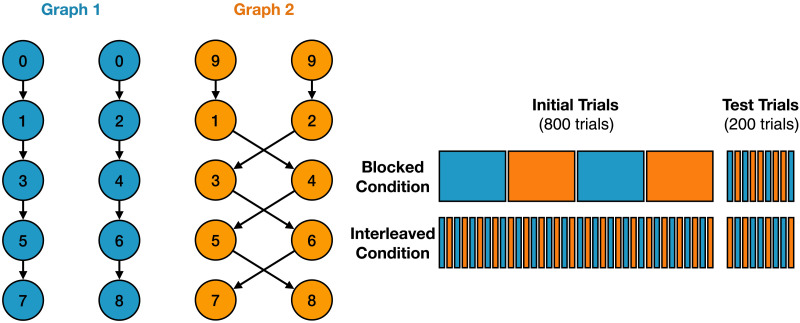
**Next-state prediction task (Beukers, Collin, et al., 2024) used for Study 2.** *Left*: Stimulus sequences for the two graphs, each consisting of two trajectories that were orthogonal across graphs. *Right*: During an initial phase, participants were exposed to sequences based on each graph, organized in either blocks of a single graph (top) or interleaved between graphs (bottom); during a test phase, all participants then completed a final randomly interleaved set of sequences.

All participants received 800 initial training trials and 200 test trials. Half of the participants received *blocked* training and the other half received *interleaved* training. In blocked training, a fixed number of sequences were randomly sampled from the same graph (with a full pass through all states in each sequence), after which the graph switched. This occurred periodically with alternating graphs (i.e., 40 sequences from graph 1, followed by 40 sequences from graph 2, then 40 from graph 1, etc.). In the interleaved version, sequences strictly alternated between the two graphs (i.e., sequence from graph 1, followed by a sequence from graph 2, then a sequence from graph 1, etc.). Finally, at the end of the experiment, all participants completed a series of sequences sampled randomly from the two graphs. This allowed for a direct comparison of performance of participants trained in the different conditions.

### Model

To simulate performance in the next-state prediction task, we augmented the model used in Study 1 by replacing linear integration in the context layer with a recurrent neural network layer that learns, using the backpropagation algorithm (Rumelhart et al., 1986), how to update its context representation^2^. This consists of a “forward pass” associated with processing (Figure 7, left side) and a “backward pass” associated with learning (Figure 7, right side). In the forward pass, on every timestep the model first calculates its updated representation of context by recurrently integrating the previous context and the current state. It then predicts the next state by querying episodic memory using the current state and the updated context. In the backward pass, the model observes the true next state, calculates a prediction error, and uses the backpropagation learning algorithm to update the weights in the recurrent neural network to reduce the prediction error. In other words, the model learns how to update the way it represents context to best predict subsequent states. Note that, as shown in Figure 7, the backpropagation of error stops at the context representation of the previous timestep (i.e., we truncate backpropagation instead of backpropagating fully through time) to better align with biologically plausble learning algorithms (e.g., Contrastive Hebbian Learning; O’Reilly, 1996; see Lillicrap et al., 2020 for a recent review of alternative approaches).

**Figure 7.**
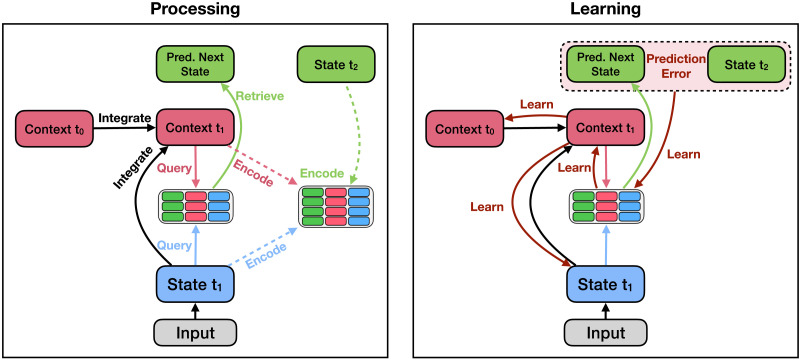
**Processing and learning for Study 2.** The model first passes the stimulus through its recurrent context layer. It then uses the stimulus and the updated context representation to retrieve a memory. It uses the retrieved memory to form a prediction of the next state. It then observes the next stimulus, calculates the prediction error, and backpropagates this error through to the weights in the recurrent layer. Finally, it encodes the updated the context representation, the current stimulus, and the successor stimulus as a new episodic memory.

After processing and learning on the current timestep, the model stores the state, context, and observed next state into episodic memory. We simulated learning and performance in the next-state prediction task by providing the model with the same sequences of stimuli presented to human participants (see Supplementary Information for details).

### Results

We measured the accuracy of the model’s predictions over time separately in the blocked and interleaved conditions (see Supplementary Information for details) and compared this to the performance of human participants and the performance of a standard recurrent neural network (an LSTM, without episodic memory) in the corresponding conditions reported by Beukers, Collin, et al. (2024). Figure 8 shows that, like human participants, the model exhibited near-ceiling performance in the blocked condition but near-chance performance in the interleaved condition. Interestingly, this obtained even in the final portion of the experiment, which involved a series of interleaved trials. That is, performance on interleaved trials was nearly *perfect* when this followed training in the *blocked* condition, but remained close to chance despite prior training on interleaved trials in the final interleaved condition. In contrast, a standard recurrent neural network fails to capture this pattern of behavior, exhibiting catastrophic forgetting under blocked training (demonstrated by the accuracy dropping to 0% on every block transition and 50% at the start of interleaved training; see Beukers, Collin, et al., 2024 for a more detailed analysis) and high performance under interleaved training.

**Figure 8.**
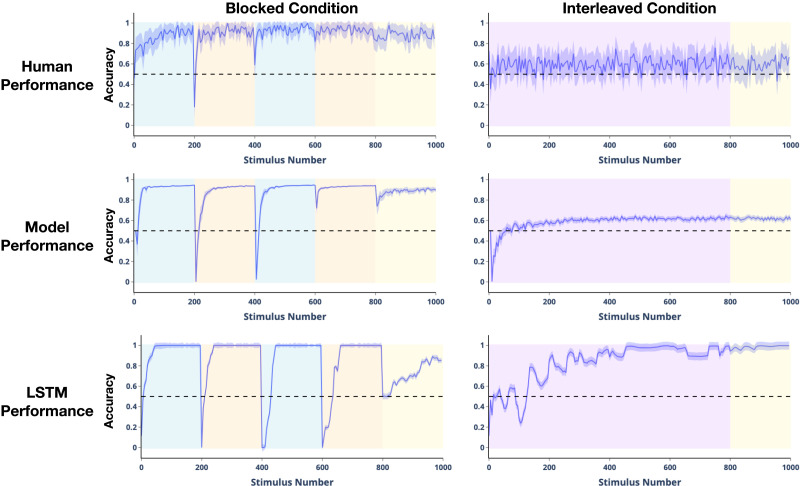
**Results for Study 2.** Accuracy for the next-state prediction task over time. In the blocked condition, the model and humans both maintain near-ceiling performance throughout the experiment, including during the final set of interleaved trials (yellow shading). In the interleaved condition, however, performance remains near-chance. In contrast, an LSTM network (without episodic memory) shows the opposite effect: in the blocked condition, the LSTM is at chance performance during the start of the interleaved trials, while in the interleaved condition, it reaches near-ceiling performance halfway through training.

To examine why the model exhibits this pattern of performance, we plotted the cosine similarity between the model’s context representations for every state against every other state. Figure 9 shows the results for training in the blocked condition. Whereas there was little structure during exposure to the first block, clear structure emerged quickly during the second block that mirrored the structure of the experiment: within each block, context representations were similar (cosine similarity close to 1; white squares in Figure 9), whereas across blocks they differed (cosine similarity less than 0; black squares squares in Figure 9). The same pattern was evident, but at a finer grain, during the final set of interleaved sequences. This pattern of results was not observed for training in the interleaved condition (see Supplementary Information).

**Figure 9.**
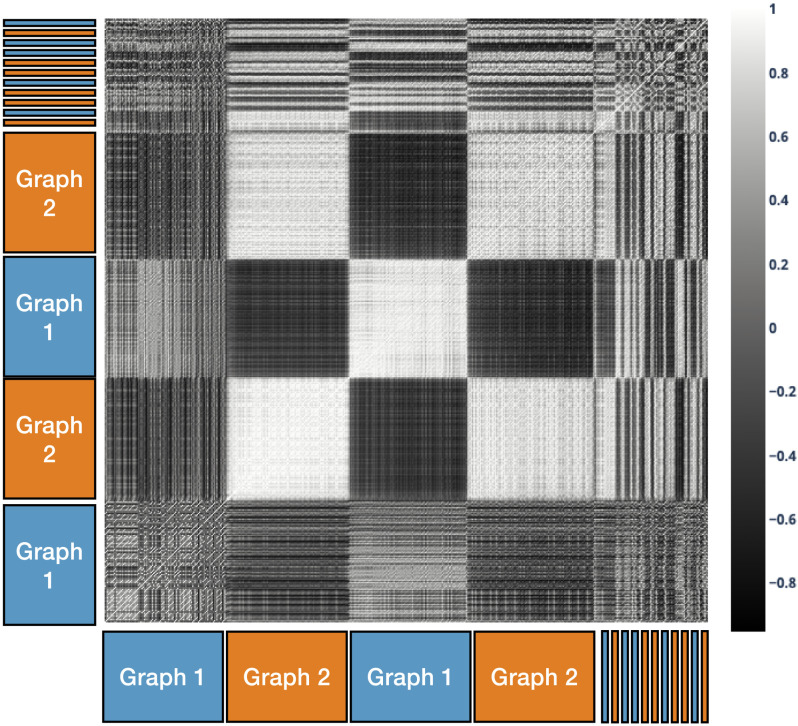
**Cosine similarity of context representations in the blocked condition of Study 2.** Shading indicates the cosine similarity between the context representation for each stimulus presentation with every other over the course of the experiment, on a scale of 1 (perfectly correlated, white) to −1 (perfectly negatively correlated, black). During the initial blocked sequences, representation similarity quickly comes to reflect the blocked structure of the experiment. During the final interleaved trials, representation similarity alternates, suggesting that context representations encode the currently active graph. This structure is not seen in the interleaved condition (see Supplementary Information).

These observations suggest that, in the blocked condition, the model learned distinct (approximately orthogonal or anti-correlated) context representations for each graph that were activated and persisted over the period during which each was relevant. To test this more directly, we used a logistic regression classifier to determine how well the pattern of activity over the context layer predicted the current graph when trained on the blocked sequences and tested on interleaved sequences at the end of the experiment. This classified the active graph in the test sequences with 88.5% accuracy (±.005 s.e.m. across the 58 model instances; significantly above chance performance, *p* < .0001 by *t*-test), supporting the hypothesis that the context representations encoded the currently active graph. For models trained in the interleaved context, however, logistic regression classified the active graph for the test sequences with only 59.5% accuracy (±.0065 s.e.m.)—significantly below the performance of the blocked model (*p* < .0001 by unpaired *t*-test)—suggesting that the model failed to learn the underlying structure of the task as effectively when trained in an interleaved manner.

### Discussion

The results suggest that a neural network architecture augmented with episodic memory can, like people, exploit blocked structure during learning to rapidly distinguish between different contexts and use representations of these contexts to control behavior accordingly. Conversely, standard neural networks without episodic memory are unable to explain these results (Beukers, Collin, et al., 2024; see Supplementary Information for a reproduction of these results): they suffer from catastrophic interference (McCloskey & Cohen, 1989) on block transitions, with learning in the new block overwriting conflicting information that was learned in the previous one. This can be avoided through interleaved training (McClelland et al., 1995), though that is the opposite of what was observed for human performance. The EGO model resolves this by rapidly learning distinct context representations for each block and storing these context representations with memories of the states in each block, allowing the model to quickly retrieve those memories and reinstate their associated context when they become relevant again. Early in training, prediction error drives the change in context, but after sufficient experience the model learns to use information from the environment (e.g., retaining the identity of the previously visited state, which disambiguates the currently active graph) rather than relying on prediction error for the shifts, allowing the model to perform well on the interleaved trials at the end of the experiment.

The behavior of the model aligns closely with CLS theory, providing an example of how the use of episodic memory allows a neural network to avoid catastrophic interference. However, the focus of CLS theory is on the rapid learning of new information that conflicts with existing knowledge stored in *semantic* memory (e.g., a penguin is a bird that cannot fly). This is accomplished by storing new information in episodic memory, keeping it separate from semantic memory, and then gradually consolidating it in semantic memory through interleaved training of information retrieved from episodic memory with existing knowledge retrieved from semantic memory (often referred to as “replay”). In the next-state prediction task, however, the challenge is to rapidly encode *semantic* information (i.e., the sequences corresponding to each graph) while keeping them distinct from *one another*. This is achieved through the rapid learning of context representations that are specific to each set of sequences, and storing these in episodic memory. From this perspective, the EGO framework can be thought of as an extension of the CLS theory, that incorporates a mechanism for learning representations that can be used for context-dependent control of behavior. This also addresses an important tension highlighted by CLS theory: whether to treat unfamiliar input as distinct from or related to existing knowledge (i.e., “pattern separation” versus “pattern completion,” respectively). The EGO framework suggests that the learning of context representations may play a critical role in mediating such decisions, by organizing memories to balance between-context differences (pattern separation) and within-context similarity (pattern completion).

Learning to group memories by context is also related to state space abstraction in reinforcement learning: The context representations learned by the model can be thought of as part of two distributed abstract state representations, corresponding to each of the two graphs latent in the structure of the environment. That is, the combination of information about the graph, which is not directly observable from the stimulus but is encoded in the currently active context representations, together with information about the current stimulus, that is encoded in the embedding layer and used to query episodic memory, allows the system to make context-appropriate inferences about the state transitions. Notably, by internally maintaining information about the environment that is not directly available, but is necessary for decision making, the context representations come to encode multi-step (non-Markovian) dependencies that convert a partially observable Markov decision process (POMDP, which is not tractable to learn) into a standard MDP (which is tractable to learn; Peshkin et al., 1999). Prior work has shown that, for short sequences, maintaining each prior state or a simple integral of those prior states in working memory may be sufficient to convert a POMDP into an MDP (e.g., Todd et al., 2008), as we demonstrated in Study 1. However, for longer or more complex sequences, using a neural network to learn compressed and/or factorized representations of prior state information can be useful (e.g., Hochreiter & Schmidhuber, 1997; Kriete et al., 2013; Rougier et al., 2005), as demonstrated in Study 2. The EGO framework integrates these approaches, providing an example of a neurally and psychologically plausible process model for state space abstraction,^3^ and the use of that information to convert POMDPs into MDPs, to solve prediction tasks using error-driven learning.

The solution to the next-state prediction task learned by the EGO model is similar to ones proposed by Beukers, Collin, et al. (2024) and Lu et al. (2023). The latter uses a neural network to learn the transitions from each state to the next, coupled with an LCI mechanism to infer and assign new task representations when prediction error signals a change in graph. The LCI-based assignment and retrieval of the representations for each graph is similar in many respects to the similarity-based retrieval of context representations from episodic memory in our model, and can be seen as implementing a similar form of state space abstraction. Critically, however, the LCI process assigns an arbitrary context representation when it infers a new graph, that is biased to be independent from previously assigned context representations. In contrast, the context representations in our model were learned *de novo*, and evolved to be dissimilar under the sequential structure of the task and the similarity-based retrieval mechanism used to retrieve them from episodic memory. From this perspective, the EGO model can be seen as offering a neurally as well as psychologically plausible implementation of the more abstract LCI model.

Finally, the context representations learned by the model can be viewed as implementing a form of cognitive control by representing and maintaining task-relevant information (in this case, a representation of the currently active graph) and using that information to guide processing (both by coordinating the retrieval of context-relevant memories and by using those memories to govern performance). In this respect, the EGO framework suggests how a recurrent neural network that represents context, coupled with the storage and similarity-based retrieval of such representations in episodic memory, can explain how control representations emerge through interactions with the environment and learning. This connects the use of context for episodic memory retrieval (e.g., Howard & Kahana, 2002; Polyn & Kahana, 2008) with the use of context for biasing processing in semantic memory (e.g., Giallanza et al., 2023), as in other models of cognitive control. We explore these interactions further in the next study.

## STUDY 3: CATEGORY LEARNING AND THE SHAPING OF REPRESENTATIONS USED FOR CONTROL

The previous experiments demonstrated a mechanism for rapidly learning context representations that can control behavior by retrieving context-appropriate episodic memories. Here, we examine how these mechanisms can interact with the learning of categories in semantic memory, shaping the formation of control representations that can flexibly select task-appropriate mappings between inputs and outputs. Specifically, we show that the model can explain results reported in a study by Flesch et al. (2018) in which, consistent with the results of Study 2 above, blocked training led to improved performance in a category learning task.

### Rationale

Prior work has suggested two important ways in which context representations can bias processing in semantic memory in support of their role in control. First, they can directly facilitate responding to task-relevant stimulus features (e.g., naming the color of a Stroop stimulus; Stroop, 1935) when such responses face competition from otherwise stronger ones (e.g., reading the word in a Stroop stimulus), by selectively biasing the activation of those features (Cohen, 2017; Cohen et al., 1990; Desimone & Duncan, 1995). Second, they can help shape the structure of semantic representations as these are being *learned*, emphasizing task-relevant distinctions between features to make these easier to selectively activate in the future (Giallanza et al., 2023; Rogers & McClelland, 2004, 2008). While previous work has addressed the use of context representations for control in these ways, little work has addressed how such context representations are *themselves* learned. Study 2 suggests that episodic memory may facilitate the rapid development of such representations, which may in turn help shape the development of semantic representations as they are formed for newly learned tasks. From this perspective, the same environmental factors that influence the learning of context representations useful for distinguishing *sequential* structure (as in Study 2) may have similar effects when learning to distinguish *feature*-based category structure.

A recent study by Flesch et al. (2018) provided support for this, using a simple feature-based category learning task. In the Flesch et al. (2018) study, participants were tasked with learning how to categorize the same set of stimuli according to one of two different features, depending on the context. Consistent with the Beukers, Collin, et al. (2024) study, participants exhibited superior performance when training was blocked by context than when it was interleaved. Subsequent work has provided evidence, from a neuroimaging experiment (Flesch et al., 2022) and a computational model (Flesch et al., 2023), that this difference in performance can be attributed to the influence the training regime had on the structure of the semantic representations that were learned: under blocked training, the semantic representations better reflected the feature relevant to the current context, whereas under interleaved training, the semantic representations reflected both features in both contexts. Flesch et al. (2023) proposed a model of these results that used a Hebbian mechanism for learning and activating context representations that decayed exponentially with time. Here, we show that the same mechanisms used in Study 2 to account for the learning of sequential structure can reproduce the results of Flesch et al. (2018) for learning feature-based category structure.

### Behavioral Task

**Figure 10.**
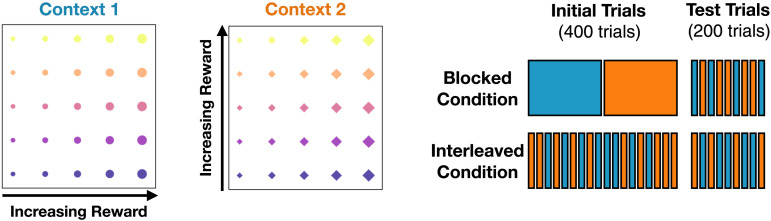
**Category learning task (Flesch et al., 2018) used for Study 3.** *Left*: Stimuli used in each of the two contexts. Stimuli varied along two abstract features, which are represented here with color and size, in two different contexts, which are represented with shape (the abstract features used in our simulation correspond to visual features shown to participants in the original study; see Supplementary Information for details). *Right*: During an initial phase, participants were exposed to sequences based on each context, organized in either blocks of a single graph (top) or interleaved between contexts (bottom); during a test phase, all participants then completed a final randomly interleaved set of sequences.

We simulated the category learning experiment described in Flesch et al. (2018), which tested the ability to learn which feature of a stimulus lead to reward in different contexts (Figure 10). Participants were shown a series of displays, each of which contained a target stimulus that varied along two feature dimensions overlaid on one of two background images. The latter reliably indicated which of the two feature dimensions determined whether the stimulus was categorized as good (positive reward) or bad (negative reward). Participants had to decide whether or not to accept or reject each stimulus (by predicting whether it would be positively or negatively rewarded), after which they were provided feedback indicating the outcome for that stimulus. Participants were not informed about the relevance of the stimulus features nor the meaning of the background images, which they had to learn through experience with the task.

Successful performance on the task required identifying which feature was relevant in each context. The design is similar to ones often used in studies of category learning (Gluck & Bower, 1988; Smith et al., 1974), Bayesian inference (Gershman et al., 2010), and reinforcement learning (Leong et al., 2017; Niv et al., 2015). Here, the focus was on how blocked versus interleaved training influenced the ability to learn the relevant feature dimension for each context and appropriately categorize the stimuli. Half of the participants completed a blocked version (in which the context switched every 200 trials) while the other half completed an interleaved version (in which the context switched randomly from trial to trial), with all participants completing an interleaved test phase at the end of the experiment.

### Model

**Figure 11.**
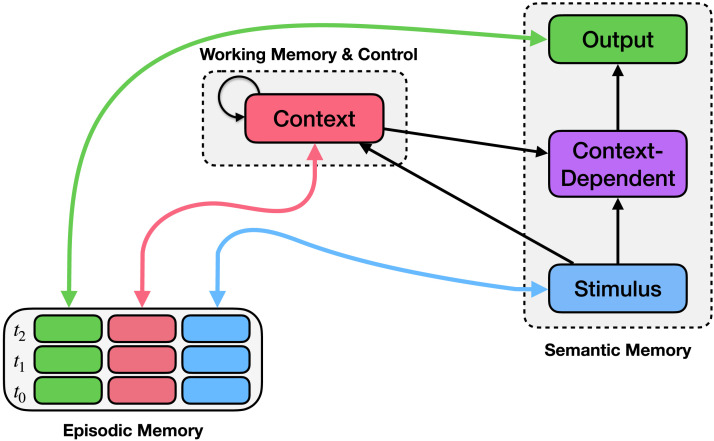
**The EGO Framework for Study 3.** The framework from Figure 1 is augmented with a context-dependent layer in the Semantic Memory module. The context-dependent layer is a neural network layer that receives input from both the stimulus and the context and projects to the output layer. This allows the context-dependent layer to represent context-relevant features of the stimulus and use those features to predict a context-appropriate output associated with the stimulus (following Rogers & McClelland, 2004). The output can thus be predicted from one of two pathways: either through episodic memory, by querying memory using the current context and stimulus, or through semantic memory, by mapping the context and the stimulus to the output layer directly using the context-dependent layer.

To simulate the task, we augmented the model used in Study 2 with a *semantic memory* pathway that adds a context-dependent layer directly connecting the stimulus and context to the output. This follows the architecture used in previous neural network models of control (e.g., Cohen et al., 1990) and semantic memory (e.g., Rogers & McClelland, 2004), as well as recent work exploring the relationship between semantics and control (Giallanza et al., 2023). Thus, the model had access to both an episodic pathway (as in Studies 1 and 2) and a semantic pathway that it could use jointly to predict the category for a given stimulus. This fully implemented the EGO architecture is shown in Figure 11. Backpropagation learning was used to update weights in all pathways (i.e., the weights connecting the stimulus, context, context-dependent, and output layers together, as well as the recurrent weights within the context module), allowing the model to learn how to extract context from the environment (episodic pathway) and how to use this context to bias processing (semantic pathway).

### Results

**Figure 12.**
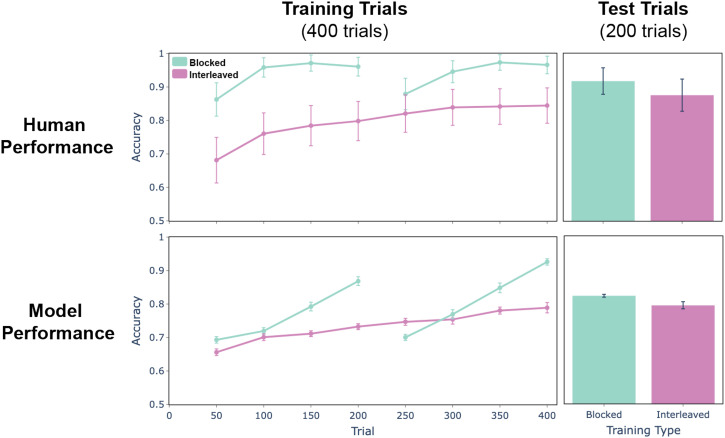
**Results for Study 3.** Accuracy in predicting positive versus negative reward during the training and test trials. During training (left panels), both humans (top) and the model (bottom) learned faster when training was blocked by context (teal) than when it was interleaved (pink). This advantage persisted during the interleaved test trials at the end of the experiment (right panels).

We trained the model to perform the task using the same protocols (blocked and interleaved) used to train participants in the Flesch et al. (2018) study (see Supplementary Information for more details). We then measured the change in accuracy over time in predicting whether the reward was positive or negative and compared performance with that of participants in the blocked and interleaved conditions of the empirical study (Figure 12). As in Study 2, both humans and the model learned to perform the task faster and better in the blocked training condition, and both performed better on interleaved test trials following blocked training, though performance was well above chance for both conditions (model: 82.2% ± 0.4 s.e.m. for blocked training and 79.3% ± 1.0 for interleaved training; humans: 91.8% ± 4.0 s.e.m. for blocked training and 87.6% ± 4.8 for interleaved training).

**Figure 13.**
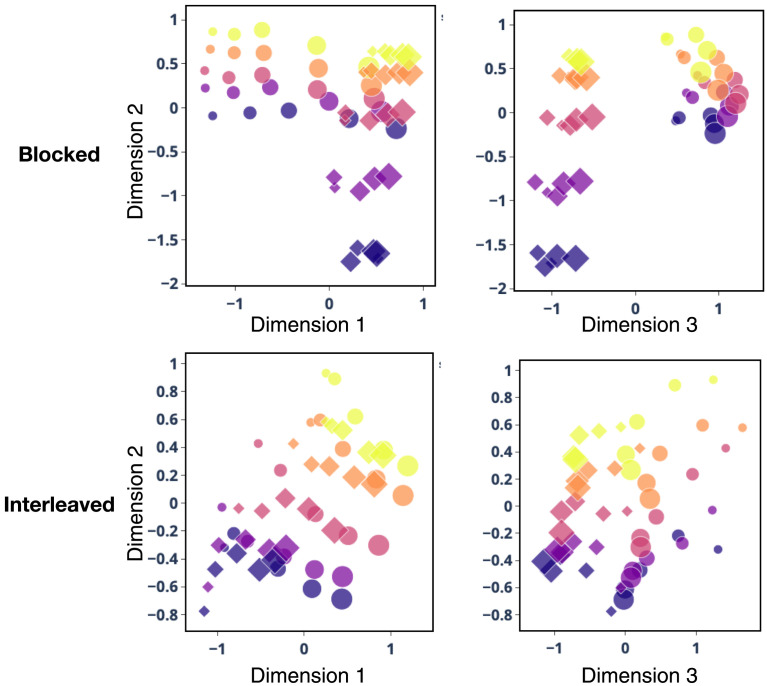
**Semantic representations learned under blocked and interleaved training in Study 3.** MDS of representations learned in the context-dependent layer of the model. Each point indicates a stimulus, with size showing the feature value along dimension 1 (*x*-axis in left panels), color the value along dimension 2 (*y*-axis in all panels), and shape designating whether it was shown with a context cue indicating whether dimension 1 (circle) or dimension 2 (diamond) predicted the outcome (*x*-axis in right panels). Training in both conditions lead to representation of the values along both stimulus dimensions. However, the blocked condition (top panels) lead to representations that were nearly orthogonal for the two categories, with low variance along the contextually-cued, task-relevant dimension (e.g., dimension 2 for the circle task, and dimension 1 for the diamonds task), and high variance along the other, task-irrelevant dimension. This was not so for the interleaved condition (bottom panels), in which representations of stimuli appear to be correlated across dimensions.

To examine the structure of the representations learned by the network, and how this affected performance, we used multidimensional scaling (MDS; Shepard, 1962) to visualize the representations that developed in the context-dependent layer (Figure 13). Each point corresponds to one stimulus, with size and color indicating the two stimulus dimensions and the shape of each point (circles versus diamonds) indicating which context was paired with the stimulus (i.e., indicating which feature dimension predicted the outcome). In both conditions, the representations clearly encode the two dimensions in an ordered way. However, in the blocked condition the variance along the irrelevant dimension is collapsed, with the circle task showing high variance for one dimension but low variance for the other, and conversely for the diamond task. In other words, following blocked training, the context-dependent layer strongly represented the task-relevant feature and only weakly represented the task-irrelevant feature, which facilitated making task-relevant responses, consistent with prior models of cognitive control (Cohen, 2017; Cohen et al., 1990; Desimone & Duncan, 1995). In contrast, in the interleaved condition the representations appear to reflect both task-relevant and task-irrelevant features equally strongly, resulting in decreased performance when these features conflicted (i.e., when the same stimulus was associated with different responses in the two contexts).

**Figure 14.**
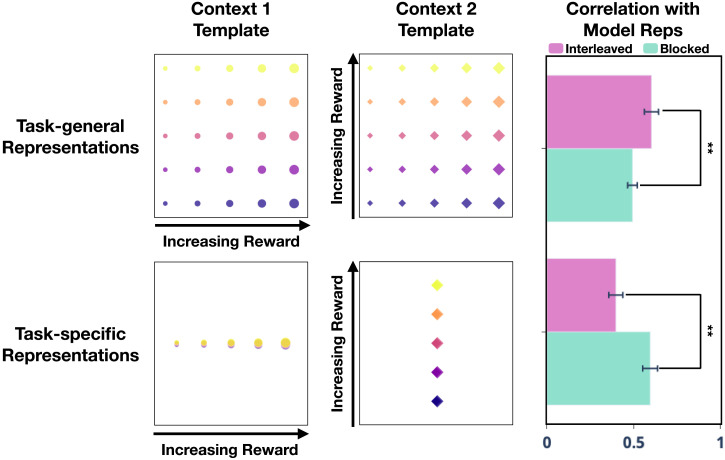
**Task-general versus task-specific representations in blocked versus interleaved training of Study 3.** Idealized representational templates (left panels) for task-general representations (top) and task-specific representations (bottom) were compared to model representations in the context-dependent layer learned under blocked and interleaved training. RSA correlations (right panel) indicate that interleaved learning (pink) favored the development of task-general representations, whereas blocked learning (teal) favored task-specific representations.

We used representational similarity analysis (RSA; Kriegeskorte et al., 2008) to quantify whether the semantic representations learned in the blocked condition more strongly reflected the task-relevant feature. Specifically, we compared the semantic representations learned in the two conditions to two idealized templates: a task-specific template, which represented only the task-relevant feature, and a task-general template, which represented both features equally strongly (Figure 14). As predicted, blocked training was more strongly associated with learning task-specific representations than was interleaved training (blocked: *r* = 0.58; interleaved: *r* = 0.39; blocked is significantly greater than interleaved by Mann-Whitney *U* test: *U* = 305, *p* = .005), whereas interleaved training was more associated with learning similar representations across tasks than was blocked (blocked: *r* = 0.51; interleaved: *r* = 0.60; blocked is significantly less than interleaved by Mann-Whitney *U* test: *U* = 97, *p* = .006).

### Discussion

The results of this study show that the interactions between episodic memory and control lead to the formation of distinct context representations for different tasks when learning to categorize based on task-specific *feature* dimensions of the stimuli, extending the results of Study 2, which focused on the learning of task-specific *sequences*. In both cases, this was promoted by blocking trials according to task, which enhanced the similarity of context representations stored in episodic memory for a given task and their differences across tasks. When the block switched, retrieving recent memories from episodic memory associated with the previous (now incorrect) context produced prediction errors that pressured the learning of distinct context representations dedicated to each task (as in Study 2).

The current results also show that the structure of these context representations impacted the structure of the *semantic* representations in the context-dependent layer of the model during blocked training by providing distinct biasing signals to that layer. This helped the semantic representations to selectively emphasize the task-relevant feature while minimizing the task-irrelevant feature (upper panels of Figure 12), ultimately improving performance on the task. This finding is consistent with prior work studying the influence of context representations used for control (e.g., Cohen et al., 1990) on the learning of semantic representations (Giallanza et al., 2023), extending those results by providing an explanation both for how context representations emerge through learning and how those can in turn shape the learning of semantic representations.

In contrast, during interleaved training the model formed context representations that were less strongly category-specific (as it did not benefit from temporal autocorrelation in the environment; see Study 2). As a consequence, the semantic representations were not as strongly biased towards selectively emphasizing task-relevant features. Instead, without a clear context signal, the model tended to learn representations that reflected both features, modulating these based on the task just enough to generate the correct response. This resulted in decreased performance, due to the potential for interference on trials in which the task irrelevant feature was associated with a different response than the task relevant one.

Finally, it is worth noting that performance of the model improved more quickly in the blocked than interleaved training condition, as is observed for human participants (see Figure 11, left panels). That is, it was faster to learn two tasks, each one-at-a-time (i.e., blocked), than it was to learn both tasks at the same time (i.e. interleaved). This might seem to run counter to the finding in the traditional literature on learning and memory that “spacing” rather than “massing” of study improves performance (Austin, 1921; Bahrick & Phelphs, 1987), if spacing is likened to interleaved and massing is likened to blocked training. However, this may be a false alignment as the effects may pertain to different timeframes of learning. One account for why spacing is better than massing is the time it affords for consolidation, because the periodic replay of information in episodic memory improves its transfer to semantic memory. The studies we have considered take place in a single session, with little if any time for such consolidation. Accordingly, the mechanisms in our model are meant to address learning on a much faster time scale, one ordinarily linked with the formation of associations in episodic memory, but that here we extend to include semantic memory.

Another factor that may differ between the studies of massing versus spacing and blocked versus interleaved training is the nature of the associations being formed. Traditionally, materials used in studies of massed versus spaced learning have not generally involved stimuli that are similar but associated with conflicting responses (i.e., incongruence of stimulus-response mappings), and thus are less likely to produce interference. Accordingly, even if spacing is akin to interleaving, performance should not be impaired due to the absence of interference effects, compared with the materials used in the studies considered here, in which the response mappings across tasks were strong potential sources of interference in the interleaved conditions.

In combination, the consideration of spacing effects suggests that future work should investigate applying the EGO framework to situations involving longer timescales of learning and tasks that do not directly conflict with one another. For example, it should be possible to train a model to learn tasks from all three studies used in this article. We would expect that the model can learn to perform all three tasks, but that spacing would become more advantageous because, on the one hand, the lack of cross-task interference limits the drawbacks of interleaved training, and, on the other hand, increasing the total amount of information that needs to be learned necessitates consolidation into semantic memory (because increasing the length of episodic memory makes it more difficult for the model to recall old memories; e.g., Beukers, Hamin, et al., 2024).

## GENERAL DISCUSSION

### Summary

One of the great remaining challenges facing the study of intelligence in natural systems, and its design in artificial ones, is understanding how the human brain achieves its (still) unique combination of efficiency of learning and flexibility of processing. These are arguably core features of its capacity for intelligence, that have been the focus of work both on semantic cognition and cognitive control, and that have yet to be replicated in artificial computational systems. Here, we argue that these capabilities reflect a close interaction among four fundamental components of cognitive function: i) statistical learning, usually attributed to semantic memory; ii) rapid storage and similarity-based retrieval of novel information, usually attributed to episodic memory; iii) activation, online integration and maintenance of recently presented information, usually attributed to working memory; and iv) use of the latter for biasing task-relevant processing, usually attributed to cognitive control.

We implement these functions in an interacting set of neural network mechanisms and describe models using these that simulate human performance in three different tasks domains that have been treated as probing largely distinct processes and explained using three different formalisms: i) revaluation in reinforcement learning, previously explained in terms of successor representations; ii) event segmentation in sequence learning, previously explained in terms of latent cause inference; and iii) the formation of context representations used for control over semantic representations in category learning, previously explained in terms of auto-associative learning mechanisms.

Here, we show how a set of integrated mechanisms can provide a single, unifying account of performance in these three domains, controlling behavior by maintaining and updating a representation of the current context (in working memory), and using this context both to recall context-relevant memories (in episodic memory) and to bias processing in favor of context-relevant features and responses (in semantic memory). We demonstrated how the interaction of episodic memory with standard mechanisms for learning and control in neural network architectures enables the system to rapidly learn new tasks without forgetting how to perform previously learned ones, reproducing effects observed for humans in revaluation, segmentation, and categorization tasks. More broadly, we demonstrated how these components, which we refer to as the EGO framework, can promote the efficient learning of abstract context representations that support flexible generalization and the optimization of control. In the remainder of this General Discussion, we consider central features of this framework, and principles it illustrates, that we organize under two broad categories: the dynamics of context processing and the structure of the representations involved.

### The Dynamics of Context Processing

#### Temporal Autocorrelation and Context Representations.

A critical factor that influenced the results is the temporal structure of the environment. We showed that models performed better following blocked training than interleaved training, in alignment with human behavior and in contrast with standard neural networks without episodic memory, which show the opposite pattern. The temporal autocorrelation present in the blocked condition benefits the models due to recurrence in the context layer. As demonstrated in Study 1, recurrent integration of stimuli induces useful structure in the context representations, such that the closer stimuli are in time to one another, the more likely it is that the context representations for those memories will be similar to one another. When related memories occur in sequence, as in blocked training, this integration, and its interaction with similarity-based retrieval from episodic memory, helps the model develop a coherent, stable representation for each context and retrieve its memories in a task-appropriate manner. This builds upon prior models of episodic memory, such as the Temporal Context Model (Howard & Kahana, 2002); but here the effect is amplified by learning, which extends its effects to longer sequences (as shown in Study 2) and to the shaping of context and semantic representations (as shown in Study 3). In contrast, during interleaved training the rapidly shifting context makes it more difficult for the models to distinctly represent the different contexts, making them susceptible to the effects of cross-task interference.

#### Task Switching and the Stability Versus Flexibility of Cognitive Control.

In addition to effects on learning, blocked versus interleaved designs may also impact the dynamics of switching between tasks, which has been a longstanding focus of the literature on cognitive control. For example, an ubiquitous finding is that rapidly switching between different control-demanding tasks harms performance, which is commonly referred to as the switch cost (Allport et al., 1972; Monsell & Mizon, 2006). This parallels the deleterious effects of interleaved designs observed in Studies 2 and 3. However, because task switching experiments generally involve highly familiar tasks (e.g., responding to the magnitude versus parity of a digit), they have not generally been thought to reflect learning (c.f. Brown & Braver, 2005; Verguts & Notebaert, 2008). Rather, they are thought to arise from difficulties in rapidly changing the allocation of cognitive control required to perform each task, reflecting a fundamental tradeoff between the requirements for stability and flexibility of control (Goschke & Bolte, 2014; Musslick & Cohen, 2021): On the one hand, focusing intently on the current task advantages performance by enhancing the processing of task-relevant information and limiting disruptions from distracting information (e.g., Cohen et al., 1990); that is, it promotes the *stability* of control. On the other hand, it also limits the ability to quickly and reliability switch between tasks; that is, it diminishes the *flexibility* of control.

The stability-flexibility tradeoff, and its optimization, has been explained in terms of the dynamics of control allocation, using models in which the representations responsible for control are implemented as attractors in the recurrent layer of a neural network that represents task contexts (e.g., Braver & Cohen, 2000; Frank et al., 2001; Gilbert & Shallice, 2002; Kalanthroff et al., 2018; Musslick et al., 2019; Zipser et al., 1993). The efficacy of such context representations can be increased by deepening the attractors, which stabilizes the representations against disruption and further facilitates the processing of task-relevant information, but makes it more difficult to switch context representations when the task changes. Conversely, if context representations are made weaker (i.e., shallower attractors), switching between contexts becomes easier, but performance of each task suffers. Normative accounts of control allocation (e.g., Shenhav et al., 2013), supported by empirical evidence (Musslick et al., 2019), suggest that humans optimize this tradeoff by estimating switch frequency and representing contexts accordingly. This explains poorer performance in interleaved designs in terms of the *dynamics* of processing (and corresponding adjustments in control) rather than learning, suggesting that these may also contribute to differences in performance in the block versus interleaved conditions of Studies 2 and 3.

#### Representational Learning and the Dynamics of Context Processing.

The models presented here did not address the dynamics of processing, nor do other models that address the phenomena discussed, all of which assume that representations are consistently engaged across trials. At the same time, representational learning—a factor that has not generally been considered in studies of task switching and control—is likely to impact the dynamics of switching between context representations. For example, in models using attractors dynamics to model task switching, dissimilar contexts are likely to be represented by more distant attractors, which will impact both the efficiency and effectiveness of moving between them. Similarly, to the extent that the distinctiveness of task representations impacts the distinctiveness of task-specific semantic representations, as shown in Study 3, then this too should impact the dynamics of task switching (e.g., Musslick et al., 2023). How representational learning and the dynamics of task switching may interact is an interesting and potentially important direction for future research.

The rapid learning in the models used in Studies 2 and 3 may also be related to questions about the dynamics of context processing and learning more broadly. Specifically, in addition to the rapid learning afforded by episodic memory storage and retrieval, in the context and context-dependent layers of the model we used a learning rate that was substantially higher than is typically used to model semantic memory (e.g., Rogers & McClelland, 2004) or in most deep learning models. Although the models still showed the same qualitative effects when using lower learning rates (see Supplementary Information), without a high learning rate the models were unable to achieve human levels of performance given the small amount of training data.

The use of a high learning rate (for adaptation on short time scales) may be viewed as an unusual application of backpropagation, which is generally used with a low learning rate over many training examples to implement long-term, statistical learning. We adopted backpropagation out of computational convenience and familiarity rather than theoretical commitment; it remains an open question whether this should be interpreted as reflecting additional mechanisms for learning or online adaptation that complement those traditionally assumed for semantic memory, or should be considered as a proxy for other activity-based optimization mechanisms (e.g., Giallanza et al., 2023), including ones that have been modeled using dynamical systems and linear quadratic regulation (Ritz et al., 2022, 2023; Tang & Bassett, 2018).

#### Episodic Memory Versus Working Memory.

Finally, whereas neural network models of task switching have focused on the dynamics of context representations actively represented in working memory, the models presented here suggest that context representations can also be retrieved from episodic memory. This distinction aligns closely with the distinction between reactive and proactive strategies for control (Braver, 2012), in which reactive control refers to “just in time” engagement of task representations, presumably retrieved from episodic memory, while proactive control is assumed to rely on the activation and maintenance of task representations in working memory. The model presented in Study 1 presents and example of such retrieval of representations from episodic memory but did not address the dynamics of doing so. This complements recent work exploring interactions between episodic and working memory in the context of other tasks used to study cognitive control, such as the n-back task (Beukers, Collin, et al., 2024; Juvina & Taatgen, 2007) and prospective memory (Lewis-Peacock et al., 2016; Momennejad et al., 2021; Ritter et al., 2018). The EGO framework may be useful in providing further theoretical unification of these phenomena with those addressed in this article.

In summary, the modeling framework we have described provides a mechanistically explicit modeling environment in which to explore the interplay between episodic memory and working memory, how their interactions impact both representational learning and the dynamics of task switching, and how those interactions are impacted by the structure of the task environment (e.g., blocked versus interleaved designs). Exploring such interactions is becoming an increasingly important direction for future research on the capacity for neural architectures to achieve flexibility as well as efficiency of processing, both in cognitive science and in machine learning, to which we will return further below.

### The Structure of Context Representations

The framework we have described also helps align several lines of work that bear on the learning of context representations, from studies spanning state space abstraction in reinforcement learning and event segmentation in sequential prediction tasks, to the learning of context representations used for control of semantic structure in categorization tasks. In previous work, these have been explained using different formalisms (e.g., successor representations, latent cause inference, and Hebbian learning, respectively). All of these provide ways for exploiting temporal autocorrelation to identify recurring features of the environment—whether these involve sequential order and/or different feature dimensions—that are specific to a given task and assigning context representations that are sensitive to these. Here, we have shown how this can be served, in all of these settings, by interactions among a common set of psychologically and neural plausible mechanisms that are responsible for the storage and similarity-based retrieval of representations in episodic memory and the integration and active maintenance of context representations in working memory.

#### Context Representations and Semantics.

Importantly, in previous efforts context representations (whether assigned or learned) have been treated as distinct from one another (e.g., using localist or otherwise orthogonal representations). The same has been true for models of cognitive control, in which representations of task context are generally low dimensional and orthogonal to one another (e.g., Cohen et al., 1990; Gilbert & Shallice, 2002; Kalanthroff et al., 2018; Musslick et al., 2023). In contrast, the framework we describe here permits the learning of distributed context representations, which can have graded similarity across tasks. Study 3 illustrated this and showed how it can be impacted by the temporal structure of the environment (e.g., blocked versus interleaved training). Recent work has shown how other statistical features of the environment (e.g., coherent covariation shared among subsets of features) can impact the learning of higher level (i.e., more abstract) representations that can be used as context for control (Giallanza et al., 2023). That is, just like semantic representations are assumed to reflect the similarity structure among stimuli, so too may higher level representations used for control (learned in the context layer) reflect the similarity structure among tasks. This suggests that, in richer environments—for example, that have hierarchical structure, such as rooms within a building or subroutines of a task—the context representations learned by the model and used for control may capture such structure. This has been demonstrated for recurrent neural networks in other settings (e.g., Botvinick & Plaut, 2004). The framework we have described here suggests that episodic memory may play an important role in contributing to this capability. Interactions with episodic memory may also play an important role in promoting the learning of abstract, relational structure that can be used for generalization and transfer, a possibility to which we will return shortly.

#### Implicit vs. Explicit Representations of Context.

It is also worth noting that the models we described learned to extract relevant context information without any explicit indications of how or when to do so. In Study 2, the model learned to activate and retain in working memory distinct representations of context associated with the transition structure indicative of two different graphs, which allowed it to disambiguate subsequent stimuli and produce the corresponding task-appropriate response; and in Study 3, the model learned that the background image for each display indicated the task-relevant feature dimension, allowing it to allocate attentional control to that dimension. In both cases, the models did this without any explicit indication of which stimuli or stimulus features were the relevant ones for use as context and control. This complements other work investigating how context representations used for control emerge through learning (e.g., Flesch et al., 2023; Giallanza et al., 2023; Rougier et al., 2005), in which it is assumed the system has access to discrete, explicitly specified task cues that are provided at every time step and initially encoded separately from other stimuli. The framework we have presented offers an opportunity to explore how these two forms of learning may interact: for example, how context representations extracted initially from cues latent in the environment (as in Studies 2 and 3) may provide the basis, with experience, for learning more explicit representations of tasks and linking these to salient cues such as task instructions, as suggested by recent work at the interface of cognitive neuroscience and machine learning, that we turn to next.

#### Episodic Memory and Abstraction.

The framework described in this article sits within an emerging, broader research agenda aimed at understanding how neural network architectures, having established their ability to learn highly complex tasks, can lean to do so with the efficiency and flexibility of processing exhibited by the human brain. One direction of such research has explored the augmentation of standard deep learning architectures with “external memory.” This refers to a form of high capacity “offline” storage, the contents of which do not impact current processing unless they are explicitly retrieved, akin to the tape of a Turing Machine. Episodic memory can be thought of as a content-addressable form of external memory, in which items are retrieved based on their similarity to a cue (or “query”) used for retrieval.^4^ Recent work in machine learning has shown that integrating such forms of external memory with traditional neural network architectures, and allowing them to learn their own read and write operations, can implement a form of “Neural Turing Machine” (Graves et al., 2014) and perform tasks that are easily implemented on traditional symbolic architectures but had previously been a challenge for neural network architectures. The transformer architecture can be seen as a close variant of this approach, in which the large space of input representations serve as a (restricted) form of external store, from which information is selectively retrieved for processing using a similarity-based (“attentional”) operation (Vaswani et al., 2017).

While networks augmented with external memory, as well as transformers (and recently their combination; e.g., Packer et al., 2023), have proven to be remarkably powerful architectures, they still fall short of meeting the challenge articulated at the outset of this article: understanding the unique combination of sample efficiency and processing flexibility of human cognitive function. Current neural network architectures require exposure to orders of magnitude more data than humans to achieve comparable levels of performance in any given task domain, and no single model has yet achieved anything close to the breadth of competencies across task domains that a human can achieve.

The models described in this article suggest ways in which it may be possible to meet these challenges. They show how, under at least some task settings, the integration of episodic memory with a recurrent network can allow the system to rapidly induce task relevant context information and use it to control performance, exhibiting a rapid form of abstraction and generalization. In addition to the use of episodic memory, an important feature of the model was the use of a relatively high learning rate. This allowed it to integrate more rapidly over time which, coupled with the similarly rapid storage and retrieval of representations from episodic memory, allowed it to discover useful context representations relatively quickly. This set of interactions can be thought of as an inductive bias toward the rapid formation of abstract representations. From this perspective, the models presented here can be seen as a simple form of “relational bottleneck,” a form of architectural inductive bias that predisposes toward the discovery of abstract relational structure (Webb, Frankland, et al., 2023). Models that implement this type of bias have been shown to exhibit remarkable improvements in sample efficiency and generalization (Altabaa et al., 2023; Kerg et al., 2022; Mondal et al., 2023; Webb, Mondal, & Cohen, 2023; Webb et al., 2020). These models have generally introduced stronger forms of a relational inductive bias than those presented here (e.g., by using episodic memory to isolate abstract from domain-specific components of processing, and serve as a variable binding mechanism that bridges between them; Webb et al., 2020). Integrating such approaches within the framework described here may be a promising approach toward building models that can address more complex tasks and abstract forms of structure discussed above.

#### Hierarchical Structure, Actions, and Planning.

Finally, our approach provides a neurally and psychologically plausible account of human behavior previously associated with model-based processing. Here, we propose that such behavior reflects the use of recurrent context representations, both actively maintained in working memory and stored in episodic memory, that are used to guide performance directly (through semantic memory) as well as through similarity-based retrieval from episodic memory. In Study 2, we showed that such context representations could be learned, and that representations learned in this way were sensitive to the latent factors mediating transitions between states, representing entire sequences of states with a single abstract representation. Furthermore, through internal replay, these representations could be used to make predictions about the distal outcome of such sequences (as in Study 1). However, none of the tasks modeled here involved any sequential dependencies on decision making or action; that is, sequences in which experienced future states were impacted by current decisions or actions. Such dependencies are a critical factor of more complex and realistic environments (such as maze navigation, game playing, etc.). The extent to which models constructed within the EGO framework can learn abstract representations of action-dependent sequences remains an important direction for future research. If successful, it would provide a set of psychologically and neurally plausible mechanisms for learning coherent sequences of actions (e.g., subroutines^5^) that may be useful for generalization and planning over longer time horizons.

### Conclusions

In this article, we presented a modeling framework for addressing the role that interactions between episodic memory, working memory, and semantic memory may play in accounting for the ability to rapidly induce abstract representations of context that can be used for the flexible allocation of control. The models we presented show how this framework can be used to provide a unified account for characteristic features of human cognitive function that have previously been addressed in different task settings (reinforcement learning, event segmentation, and category learning) and explained using different formalisms (successor representations, latent cause inference, and Hebbian learning).

The framework extends a rich tradition of using neural network models to understand human cognitive functions, including the Temporal Context Model of episodic memory function (Howard & Kahana, 2002); Complementary Learning Systems theory (McClelland et al., 1995) concerning the interactions between episodic and semantic memory; as well as the modeling of cognitive control (Cohen et al., 1990) and its interaction with semantic cognition (Giallanza et al., 2023). It also provides insights into the interaction between two forms of control that have previously been proposed (Cohen & O’Reilly, 1996): rapid formation of novel associations (e.g., as provided by task instructions) through the rapid *binding* of novel information in episodic memory and the effects of context representations actively maintained in working memory in *biasing* processing in task-specific pathways (Cohen et al., 1990; Miller & Cohen, 2001).

It also builds on previous work identifying these formalisms with the function of underlying neural mechanisms: episodic memory, thought to reflect rapid associative learning in medial temporal structures (including the hippocampus; McClelland et al., 1995) and perhaps others, such as the cerebellum (Webb, Frankland, et al., 2023); the active maintenance of context representations used for control in working memory, thought to reflect the function of prefrontal cortex (Miller & Cohen, 2001) in concert with basal ganglia (Frank et al., 2001); and semantic memory, reflecting slower forms of statistical learning and distributed representations, thought to be subserved by mechanisms distributed throughout the rest of neocortex.

Finally, although the models we described were relatively simple, we have pointed out how the EGO framework in which they were developed makes contact with recent work both in cognitive science and machine learning that integrates external memory with traditional neural network architectures, and uses this as an inductive bias for abstraction, generalization, and the flexible control of behavior. These suggest potentially promising directions for extensions of the models we have described to address more complex tasks going forward.

In sum, we hope that this work provides a useful framework and examples of its application in addressing the challenge with which we began: understanding the human capacity for efficient acquisition of abstract representations that can be used for generalization and the flexible control of behavior, capabilities that are cornerstones of the human capacity for general intelligence.

## ACKNOWLEDGMENTS

The authors would like to thank Tim Rogers, Kenneth Norman, and Nathaniel Daw for valuable conversations that helped guide the work reported in this article.

## FUNDING INFORMATION

This work was carried out with support from an NSF Graduate Fellowship to TG and a Vannevar Bush Faculty Fellowship to JDC.

## AUTHOR CONTRIBUTIONS

T.G.: Conceptualization; Data curation; Formal analysis; Investigation; Methodology; Project administration; Resources; Software; Supervision; Validation; Visualization; Writing – original draft; Writing – review & editing. D.C.: Formal analysis; Investigation; Methodology; Software; Visualization; Writing – review & editing. J.C.: Conceptualization; Funding acquisition; Investigation; Project administration; Supervision; Validation; Writing – review & editing.

## DATA AVAILABILITY STATEMENT

All data and code to reproduce the simulations are available on GitHub (https://github.com/tylergiallanza/EmergentIntelligentControl).

## Notes

^1^ CLS outlines how episodic memories get consolidated into semantic memory through offline replay that interleaves new and old experiences. This explains how exceptions to general knowledge can be accommodated—e.g., by interleaving the newly learned fact that a penguin is a bird that cannot fly with the existing general knowledge that most birds fly.^2^ Following prior work (Braver & Cohen, 2001; O’Reilly & Frank, 2006), we used a gated recurrent network rather than a simple RNN (we specifically used the Minimal Gated Unit; Zhou et al., 2016). We found that the gated network performed better than the simple network across training conditions. Additionally, we used a learning rate value higher than what is traditionally used to train neural networks. This allowed the model to achieve human-level performance despite the small number of training examples. We found that the qualitative effects described in the Results section held for a variety of learning rates, however (see Supplementary Information).^3^ As noted above, the state space abstraction in the model used for Study 2 is distributed over the context layer, which serves to identify the graph, and the representations stored in episodic memory over which similarity-based retrieval is used to determine the response.^4^ This contrasts with “index-addressable” memory, in which items are retrieved based on the physical location at which they are stored. However, insofar as locations are accessed by their “address,” and storage is systematic (e.g., sequentially written to sequential addresses), then temporal context can be thought of as implementing a form of indexing within the broader scope of content-addressable memory. This corresponds closely to the use of positional encoding in transformers (Vaswani et al., 2017).^5^ In the context of reinforcement learning, this has been referred to as “option discovery” (Sutton et al., 1999), and may be an important mechanism for learning hierarchical forms of behavior and planning.
